# Spironolactone Effect in Hepatic Ischemia/Reperfusion Injury in Wistar Rats

**DOI:** 10.1155/2016/3196431

**Published:** 2015-12-21

**Authors:** Julio César Jiménez Pérez, Araní Casillas Ramírez, Liliana Torres González, Linda Elsa Muñoz Espinosa, Marlene Marisol Perales Quintana, Gabriela Alarcón Galván, Homero Zapata Chavira, Francisco Javier Guzmán de la Garza, Carlos Rodrigo Cámara Lemarroy, Nancy Esthela Fernández Garza, Edelmiro Pérez Rodríguez, Paula Cordero Pérez

**Affiliations:** ^1^Liver Unit, Department of Internal Medicine, University Hospital “Dr. José E. González”, The Autonomous University of Nuevo León, 64460 Monterrey, NL, Mexico; ^2^Hospital Regional de Alta Especialidad de Ciudad Victoria “Bicentenario 2010”, 87087 Cuidad Victoria, TAMPS, Mexico; ^3^Transplant Service, University Hospital “Dr. José E. González”, The Autonomous University of Nuevo León, 64460 Monterrey, NL, Mexico; ^4^Department of Pathology, University Hospital “Dr. José E. González”, The Autonomous University of Nuevo León, 64460 Monterrey, NL, Mexico; ^5^Department of Physiology, School of Medicine, The Autonomous University of Nuevo León, 64460 Monterrey, NL, Mexico

## Abstract

*Introduction*. Ischemia/reperfusion (IR) injury, often associated with liver surgery, is an unresolved problem in the clinical practice. Spironolactone is an antagonist of aldosterone that has shown benefits over IR injury in several tissues, but its effects in hepatic IR are unknown. *Objective*. To evaluate the effect of spironolactone on IR-induced damage in liver. *Materials and Methods*. Total hepatic ischemia was induced in rats for 20 min followed by 60 min of reperfusion. Spironolactone was administered and hepatic injury, cytokine production, and oxidative stress were assessed. *Results*. After IR, increased transaminases levels and widespread acute inflammatory infiltrate, disorganization of hepatic hemorrhage trabeculae, and presence of apoptotic bodies were observed. Administration of SPI reduced biochemical and histological parameters of liver injury. SPI treatment increased IL-6 levels when compared with IR group but did not modify either IL-1*β* or TNF-*α* with respect to IR group. Regarding oxidative stress, increased levels of catalase activity were recorded in IR + SPI group in comparison with group without treatment, whereas MDA levels were similar in IR + SPI and IR groups. *Conclusions*. Spironolactone reduced the liver damage induced by IR, and this was associated with an increase in IL-6 production and catalase activity.

## 1. Introduction

Clamping of the hepatic pedicle during resection of liver tumors or liver transplantation is often unavoidable, and during these conditions hepatic ischemia/reperfusion (IR) injury may occur. IR injury is the main cause of primary graft dysfunction or nonfunction after liver transplantation. In addition, the liver suffers from warm IR injury during tissue resections (Pringle Maneuver), hemorrhagic or endotoxin shock, and thermal injury [1]. Hepatic IR involves a complex series of processes that comprises microcirculatory failure, followed by necrosis and cell death [2]. The destructive effects of IR are in part triggered by the acute generation of reactive oxygen species following reoxygenation, which causes direct tissue injury and initiates a chain of deleterious cellular responses leading to inflammation and cell death, which eventually culminate in target organ failure [3]. Current strategies for the treatment of liver IR injury are either preventive [4] or pharmacological [5]. Pharmacological modulation may have a more universal application; however, several therapeutic formulations have been studied and none has been fully successful in preventing mortality associated with liver IR [6, 7]. Thus, the development of new strategies for prevention and treatment of liver damage due to IR is critical to improving outcomes for patients under such conditions.

Recent studies in humans and experimental models have shown that aldosterone plays a pivotal role in the pathophysiology of cardiovascular and renal injury. In this regard, clinical trials have evidenced that mineralocorticoid receptor (MR) blockade improves the survival of patients with chronic heart disease and chronic renal failure [8–11]. The protective effect of MR blockade is associated with decreased fibrosis and vascular inflammation, suggesting that aldosterone is a profibrotic hormone [12, 13]. Spironolactone (SPI) is a synthetic 17-lactone steroid, which is a competitive aldosterone antagonist in a class of pharmaceuticals called potassium-sparing diuretics. SPI is considered fourth line therapy for hypertension in patients already treated with multiple medications [14, 15]. Antagonists of aldosterone have shown beneficial effects in IR experimental models in retina [16], intestine [17], heart [18], kidney [19], and brain [20], but nothing has been reported yet in the setting of hepatic IR injury.

In this study, we sought to evaluate the effect of SPI in livers undergoing normothermic IR injury and to investigate if the protective effects of SPI could be associated with a reduction in oxidative stress and the inflammatory response.

## 2. Materials and Methods

### 2.1. Animals

Animal procedures were performed in accordance with the proper use and care of laboratory animals, approved by the ethics committee of our institution. Experiments were performed using 15 male Wistar rats weighing 200–250 g. Animals were maintained under standard conditions such as stable room temperature (24 ± 3°C) and a 12-hour light-dark cycle and were allowed access to commercial rat pellets and water ad libitum.

### 2.2. Experimental Model

Briefly, after 24 hours of fasting, the animals were anesthetized with pentobarbital sodium anesthesia (60 mg/kg i.p.) and were placed below a heating lamp to maintain constant temperature (37°C), and an identical midline abdominal incision was performed.

#### 2.2.1. Total Hepatic Ischemia

The hepatic hilum was identified and complete warm hepatic ischemia was induced by Pringle Maneuver [21, 22] with microvascular bulldog clamps; ischemia was noticed by color changes in the liver and intestinal tissue. Hepatic ischemia was maintained for 20 minutes, and then clamps were removed to allow 60-minute reperfusion, after which blood and liver samples were collected, and rats were humanely sacrificed.

#### 2.2.2. Partial Hepatic Ischemia

A model of 70% hepatic ischemia was also used, following procedures described in the literature [23]. Briefly, after midline laparotomy, the liver was freed from its ligaments and subsequently all structures of the portal triad of the left and median hepatic lobes were occluded for 60 minutes with a microvascular clamp (Aesculap, San Francisco, CA). In this model, mesenteric congestion is prevented by allowing intestinal blood flow through the right and caudate lobes. The clamps were then removed to allow 6-hour reperfusion, after which blood and liver samples were collected, and rats were humanely sacrificed.

### 2.3. Experimental Protocol

*Protocol 1 (dose-response study to assess the effect of SPI on hepatic injury)*. To determine the most effective dose of SPI in reducing hepatic injury in conditions of complete warm ischemia, a dose-response study was carried out to evaluate the effect of several doses of SPI as follows:

(1.A) IR group (*n* = 5 rats): animals were subject to total hepatic ischemia as described above and received vehicle only (saline).
(1.B) IR + SPI group (*n* = 5 rats): it is as in group B, but animals received SPI at dose of 1000 *μ*g/kg orally 20 hours before induction of IR.
(1.C) IR + SPI group (*n* = 5 rats): it is as in group B, but animals received SPI at dose of 2600 *μ*g/kg orally 20 hours before induction of IR.
(1.D) IR + SPI group (*n* = 5 rats): it is as in group B, but animals received SPI at dose of 5000 *μ*g/kg orally 20 hours before induction of IR.
(1.E) IR + SPI group (*n* = 5 rats): it is as in group B, but animals received SPI at dose of 10,000 *μ*g/kg orally 20 hours before induction of IR.
(1.F) IR + SPI group (*n* = 5 rats): it is as in group B, but animals received SPI at dose of 20,000 *μ*g/kg orally 20 hours before induction of IR.

*Protocol 2 (effect of SPI on hepatic injury associated with normothermic IR)*. To evaluate whether spironolactone treatment at the most effective dose could reduce hepatic injury in conditions of either total or partial normothermic IR, the following experimental groups were performed:

(2.A) Sham group (*n* = 5 rats): animals received only Sham surgery, where laparotomy was performed but liver was only manipulated and warm hepatic ischemia was not induced.
(2.B) IR group (*n* = 5 rats): animals were subject to total hepatic ischemia as described above and received vehicle only (saline).
(2.C) IR + SPI group (*n* = 5 rats): it is as in group B, but animals received SPI (2.6 mg/kg) orally 20 hours before induction of IR.
(2.D) PIR group (*n* = 5 rats): animals were subject to partial hepatic ischemia as described above and received vehicle only (saline).
(2.E) PIR + SPI group (*n* = 5 rats): it is as in group D, but animals received SPI (2.6 mg/kg) orally 20 hours before induction of IR.

### 2.4. Histological Examination

Immediately after obtaining the liver, the sample was fixed in 10% neutral buffered formalin. Samples were then embedded in paraffin, and 4 *μ*m thick sections were stained with hematoxylin and eosin and examined under light microscope by a blinded pathologist. The hepatic histological damage and hepatocellular necrosis were evaluated according to the Shen [24] and Chen [25] scales, respectively.

The hepatic histological damage scale consists in 4 degrees (G0–G3): grade 0 indicates minimal or no evidence of injury; grade 1 indicates mild injury with cytoplasm vacuolization and focal nuclear pyknosis; grade 2 indicates moderate-to-severe injury with extensive nuclear pyknosis, loss of intercellular borders, and mild-to-moderate neutrophil infiltration; grade 3 indicates severe injury with disintegration of hepatic cords, hemorrhage, and severe PMN infiltration. The hepatocellular necrosis scale consists in 4 degrees (G0–G3): none is grade 0, single cell is grade 1, −30% is grade 2, and >30% is grade 3.

### 2.5. Biochemical Analysis

#### 2.5.1. Measurements of Transaminases

Blood samples were used to determine serum levels of ALT and AST by standard commercial biochemical assay kits, using DT6011 analyzer (Vitros DTII Systems Chemistry, module DTSCII; Johnson & Johnson Ortho-Clinical Diagnostics, New Brunswick, NJ, USA).

#### 2.5.2. Cytokine Determination

Serum levels of tumour necrosis factor-alpha (TNF-*α*), Interleukin-1 (IL-1), and Interleukin-6 (IL-6) were determined using a rat TNF-alpha, IL-1, and IL-6 enzyme, linked immunosorbent assay (ELISA) kit (Peprotech, México).

#### 2.5.3. Oxidative Stress Parameters

In serum samples, total antioxidant capacity was determined using an Antioxidant Assay Kit, which assesses the combination of both small molecule and protein antioxidants (Cayman Chemical Company, Michigan, USA); catalase activity using a Catalase Assay Kit (Cayman Chemical Company, Michigan, USA); and malondialdehyde (MDA) using a MDA Assay Kit (Cayman Chemical Company, Michigan, USA).

### 2.6. Statistical Analysis

The SPSS 22.0 statistical software package (SPSS Inc. Software, Chicago, Illinois, USA) was used to analyze data using one-way analysis of variance (ANOVA) and Tukey's post hoc test to determine comparison between groups and differences between groups, respectively. All values are expressed as mean ± standard deviation (SD) and *P* < 0.05 was considered statistically significant. Pearson's chi-square test was applied for histological examination; *P* value < 0.05 was considered statistically significant. The dose-response study results were analyzed using Prism version 6 (GraphPad Software Inc., San Diego, CA). Data were evaluated by one-way analysis of variance and Bonferroni's post-test.

## 3. Results

### 3.1. Dose-Response Effect of SPI on Hepatic Injury in Total Warm Ischemia

**Figure 1:**
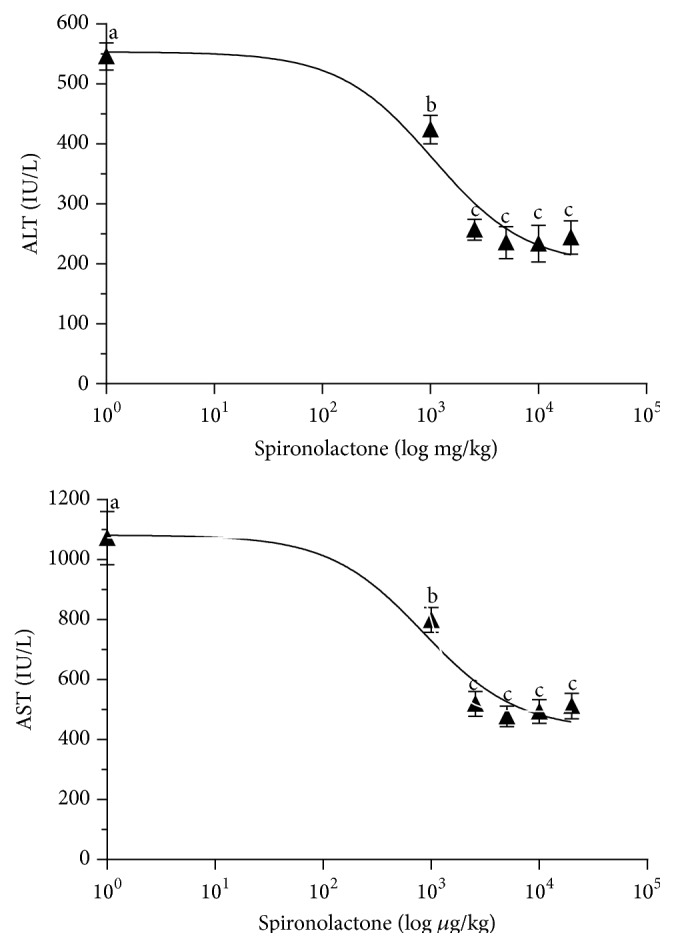
Dose-response study of spironolactone on hepatic injury in total normothermic IR. The effects of SPI treatment on ALT and AST levels were assessed. Rats were treated with SPI (0, 1,000, 2,600, 5,000, 10,000, and 20,000 *μ*g/kg). Transaminase levels were measured after 1 h of reperfusion. Means without a common letter are different; *P* < 0.05.

We administered SPI at doses of 1,000, 2,600, 5,000, 10,000, and 20,000 *μ*g/kg in rats 20 hours before the surgical procedure, and the effects on hepatic injury were determined 1 h after reperfusion. Our results indicated that SPI protected livers against damage in a dose-dependent manner. The ED50 values for ALT and AST were 1,056 *μ*g/kg and 1,030 *μ*g/kg, respectively. The most effective dose of SPI in reducing the parameters of hepatic injury in liver undergoing warm ischemia was 2,600 *μ*g/kg (2.6 mg/kg). This dose was then used in the rest of the experimental procedures. Higher doses were not associated with lower hepatic damage (Figure 1).

### 3.2. Spironolactone as Pharmaceutical Strategy to Reduce Hepatic IR Injury

**Figure 2:**
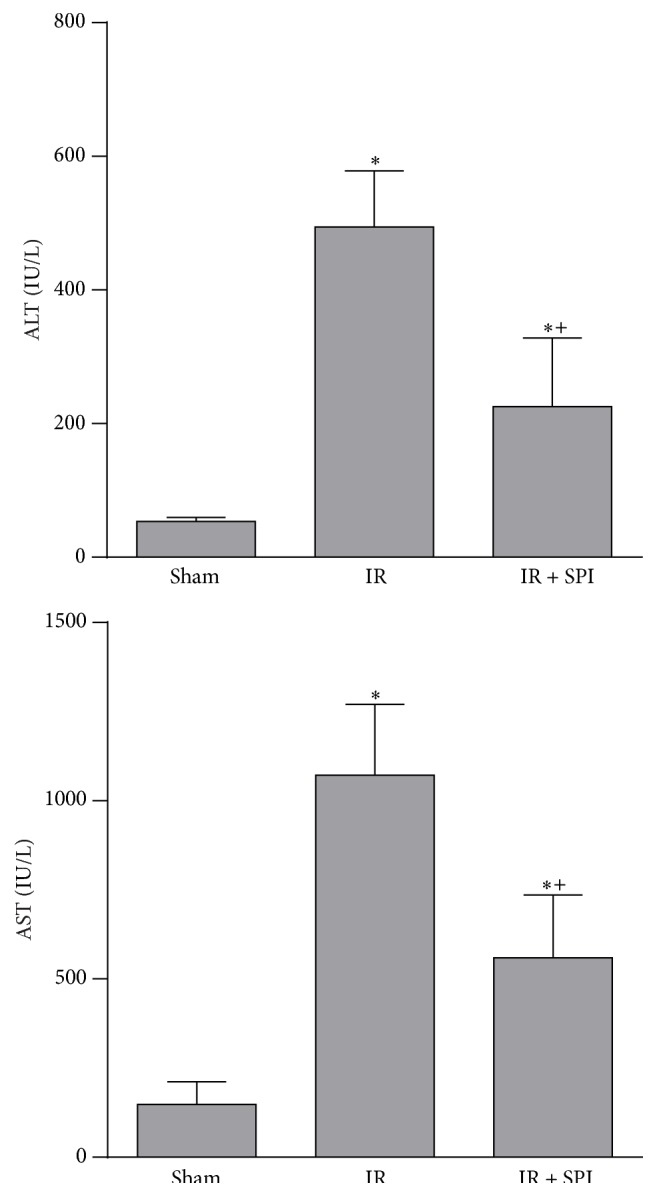
Effect of spironolactone on biochemical parameters of liver injury in total normothermic IR. ALT and AST levels were measured in plasma. ^*∗*^ *P* < 0.05 versus Sham; ^+^ *P* < 0.05 versus IR.

**Figure 3:**
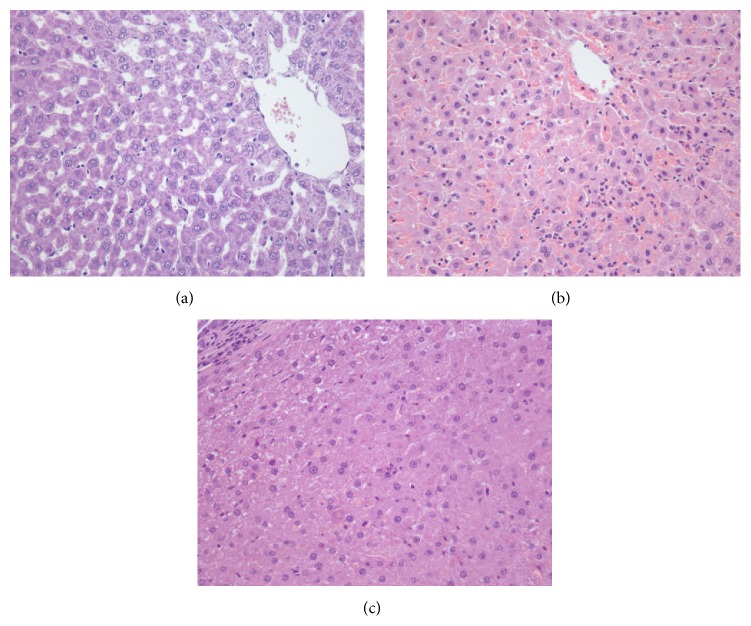
Hematoxylin and eosin staining of hepatic tissue. The Sham group (a) showed conserved cellular architecture. IR group (b) showed numerous inflammatory cell groups predominantly perivenular and presence of apoptotic bodies isolated surrounded by inflammation. The IR + SPI group (c) showed conserved cellular architecture, isolated pockets of acute inflammation, and apoptotic bodies.

In the total liver normothermic IR model, the administration of spironolactone at the selected dose of 2.6 mg/kg (IR + SPI group) reduced ALT and AST levels compared with the results obtained in IR group (ALT values: 494 ± 83.9 and 226 ± 103 IU/L for the IR and IR + SPI, resp.; AST values: 1072 ± 198 and 559 ± 176 IU/L, for the IR and IR + SPI, resp.) (Figure 2). Biochemical parameters of hepatic injury were consistent with histological study of the liver. The IR group showed extensive inflammatory infiltrate with presence of apoptotic bodies. The IR + SPI group showed conserved cellular architecture, isolated pockets of acute inflammation, and apoptotic bodies (Figure 3). Significantly lower histological damage and hepatocellular necrosis scores were found in the IR + SPI group when compared with IR group at the end of reperfusion (Table 1).

**Figure 4:**
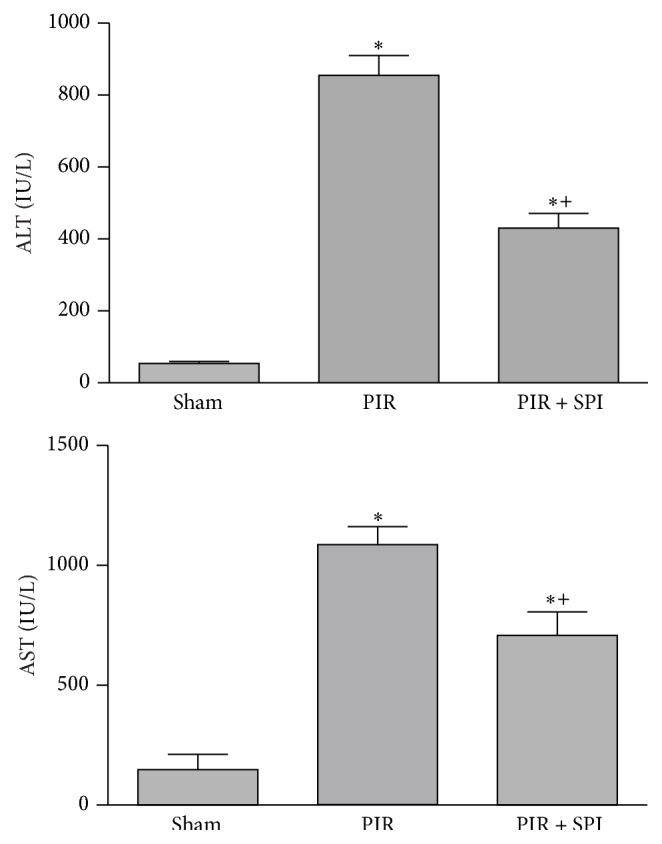
Effect of spironolactone on biochemical parameters of liver injury in normothermic PIR. ALT and AST levels were measured in plasma. ^*∗*^ *P* < 0.05 versus Sham; ^+^ *P* < 0.05 versus PIR.

Similar results were obtained in the partial liver IR model, where the administration of spironolactone at the selected dose of 2.6 mg/kg (PIR + SPI group) reduced ALT and AST levels compared with the results obtained in PIR group (ALT values: 855 ± 55 and 431 ± 41 IU/L for the PIR and PIR + SPI, resp.; AST values: 1085 ± 75 and 708 ± 80 IU/L, for the PIR and PIR + SPI, resp.) (Figure 4).

### 3.3. Effect of SPI on Cytokine Production and Oxidative Stress in Hepatic Normothermic IR Injury

**Figure 5:**
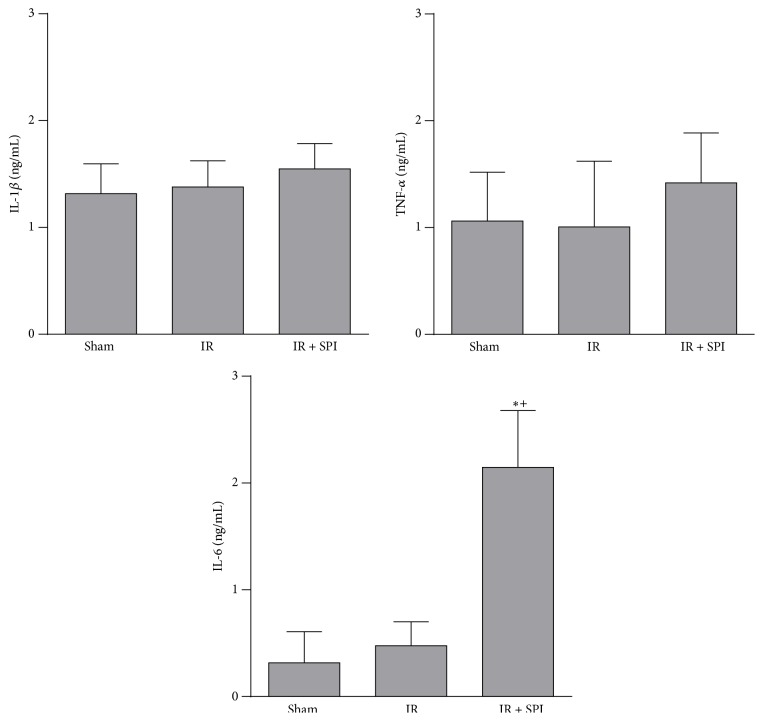
Effect of spironolactone on cytokine production in normothermic IR injury. IL-1*β*, IL-6, and TNF-*α* levels were measured in plasma. ^*∗*^ *P* < 0.05 versus Sham; ^+^ *P* < 0.05 versus IR.

As shown in Figure 5, IL-1*β*, TNF-*α*, and IL-6 levels of the IR group were of the same order as those of the Sham group (IL-1*β* values: 1.38 ± 0.25 and 1.32 ± 0.28 ng/mL, in IR and Sham groups, resp.; TNF-*α* values: 1.01 ± 0.61 ng/mL and 1.06 ± 0.46 ng/mL, in IR and Sham groups,; IL-6 values: 0.48 ± 0.23 and 0.32 ± 0.29 ng/mL, in IR and Sham groups, resp.). Treatment with spironolactone did not result in changes in plasma IL-1*β* and TNF-*α* levels with regard to those found in the IR group (IL-1*β* values: 1.55 ± 0.24 and 1.38 ± 0.25 ng/mL, in IR + SPI and IR groups, resp.; TNF-*α* values: 1.42 ± 0.47 and 1.01 ± 0.61 ng/mL, in IR + SPI and IR groups, resp.). However, IR + SPI group showed increased IL-6 levels when compared with the IR group (IL-6 values: 2.15 ± 0.53 and 0.48 ± 0.23 ng/mL, in IR + SPI and IR groups, resp.) (Figure 5).

**Figure 6:**
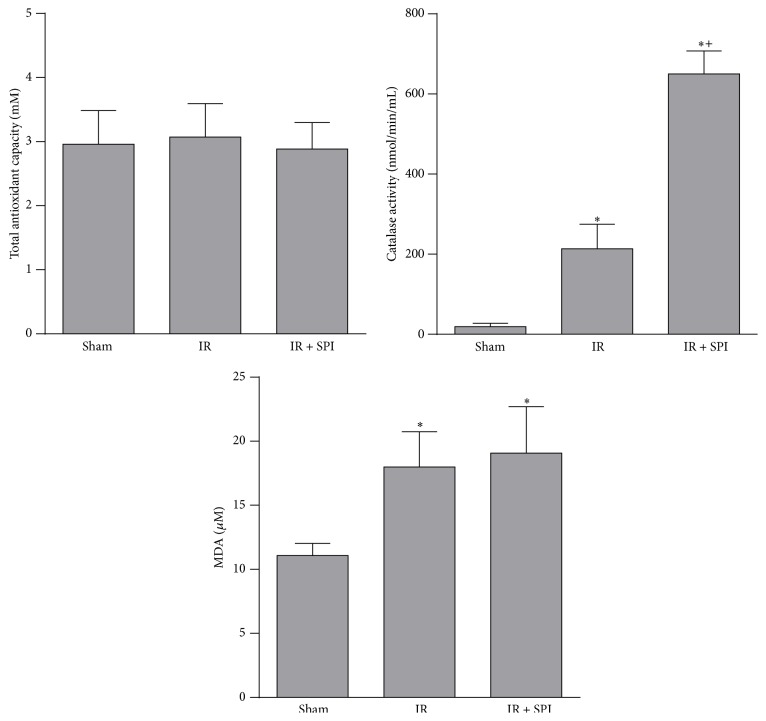
Effect of spironolactone on oxidative stress in normothermic IR injury. Total antioxidant capacity, catalase activity, and MDA levels were measured in plasma. ^*∗*^ *P* < 0.05 versus Sham; ^+^ *P* < 0.05 versus IR.

Regarding oxidative stress parameters, total antioxidant capacity of the IR and Sham groups was similar (total antioxidant capacity values: 3.07 ± 0.52 and 2.96 ± 0.52 mM, in IR and Sham groups, resp.). IR increased catalase activity and MDA levels when compared with the Sham group (catalase activity values: 214.22 ± 61.1 and 18.9 ± 8.50 nmol/min/mL, in IR and Sham groups, resp.; MDA values: 18.0 ± 2.75 and 11.1 ± 0.96 *μ*M, in IR and Sham groups, resp.). Spironolactone did not have a significant effect over total antioxidant capacity, since this parameter in the IR + SPI group was similar to that recorded in IR group (total antioxidant capacity values: 3.07 ± 0.52 and 2.89 ± 0.41 mM, in IR + SPI and IR groups, resp.). Results showed an increase in catalase activity in the IR + SPI group in comparison with the IR group (catalase activity values: 651.55 ± 57 and 214 ± 61.1 nmol/min/mL, in IR + SPI and IR groups, resp.). Treatment with spironolactone did not modify MDA levels with respect to the IR group (MDA values: 19.1 ± 3.61 and 18.0 ± 2.75 *μ*M, in IR + SPI and IR groups, resp.) (Figure 6).

## 4. Discussion

We found that SPI was able to reduce liver IR injury in total liver IR models, as evidenced by attenuation of the histopathological alterations associated with IR injury as well as by reduction of serum levels of AST and ALT. Spironolactone is widely used in clinical practice [25–27]. In fact, several studies have evaluated the usefulness of spironolactone in the treatment of ischemia reperfusion in organs such as kidney and heart in clinical studies, and the results obtained have shown beneficial effects of this drug [28–30]. Our results reveal that pretreatment with spironolactone could open new pathways for protecting liver against IR injury, a strategy that could turn out to be clinically relevant.

As the experimental model of total hepatic IR involves the fact that both gut and liver are subjected to ischemic conditions, it is possible to consider that the observed effects on the liver might thus represent gut-originating responses to ischemia and their modification by SPI. To assess this possibility, an experimental model of partial hepatic IR was carried out to evaluate the effect of SPI on hepatic injury, since in this model intestinal congestion is prevented. Our results indicated that the same dose of SPI was able to reduce biochemical parameters of hepatic injury, thus indicating a liver-specific protective effect for SPI in normothermic hepatic IR.

It is well known that, during hepatic IR, cytokines are released through the induction of adhesion molecules (ICAM and vascular cell adhesion molecule [VCAM]) and CXC chemokine which leads to neutrophil activation and accumulation. These neutrophils then extravasate, causing parenchymal injury by ROS production [31]. Several experimental studies in IR models in tissues different to liver have demonstrated that SPI has anti-inflammatory activity, which may rely on its ability to modulate the production of cytokines including IL-1*β*, TNF-*α*, and IL-6 [17, 32, 33]. By analyzing these parameters in our study, the benefits of spironolactone could be associated only with increased IL-6 production. IL-6 treatment has been found to have protective effects against warm IR injury in rodents [34]. One study showed worse IR injury in livers of IL-6 knockout mice than wild type mice, which was restored to the wild type injury patterns by administration of recombinant IL-6 to the knockout mice before ischemia [35]. These results suggest that SPI-induced protection against IR injury could be partly explained by modulation of IL-6 levels but not by any effects over IL-1*β* or TNF-*α*.

Spironolactone had a protective effect in several models of IR injury model through amelioration of oxidative stress. [16, 17]. In this study, three oxidative stress mediators were analyzed: total antioxidants, catalase activity, and MDA. Unlike other studies that have reported reduction in oxidative stress parameters after SPI administration [17, 19, 28], we found no relevant changes in MDA. This could be due to differences in the experimental conditions in the models used. Our results suggest that, in the conditions evaluated herein, SPI did not reduce markers of oxidative stress. We evaluated total antioxidant capacity but results indicated that SPI treatment did not induce any change in this parameter. Then, we decided to assess catalase activity, since this enzyme is one of the most important antioxidants in the context of hepatic IR. [25, 36]. The effect of SPI on catalase was evaluated, and we found that SPI increased catalase activity at the systemic level. Thus, in addition to inducing IL-6 production, SPI increased antioxidant enzymes, resulting in the preservation of hepatic structure and reduction of liver injury, as was shown by the light microscopic findings and the biochemical liver injury markers. This may indicate that SPI is inducing endogenous protective mechanisms in hepatic tissue as a way to counteract the injurious effects of normothermic IR.

Studies on myocardial infarction models have also shown that mineralocorticoid receptor blockers can modulate macrophage function, thus diminishing the cellular inflammatory response [37]. The proteolytic enzyme cathepsin was also shown to be modulated by MR blockade in an intestinal IR injury model [38]. Whether these mechanisms are implicated in SPI-induced hepatoprotection in liver IR injury remains unknown and deserves further studies.

There are some limitations in this study. The sample size is small, and molecular mediators were assessed on serum and not on tissue. This could reflect more a systemic response to IR, irrespective of the beneficial effects we observed over histopathological scores. Finally, further studies would be necessary to conclude that these results are clinically relevant.

## 5. Conclusions

In conclusion, the present study demonstrated for the first time that SPI has hepatoprotective properties in IR liver injury model. This effect was associated with the induction of protective mechanisms in hepatic tissue such as IL-6 production and increased catalase activity.

## Figures and Tables

**Table 1:** Evaluation of hepatic tissue according to histological damage.

	Hepatic histological damage scale	Hepatocellular necrosis scale
Sham	1	0
IR	2^*∗*^	3^*∗*^
IR + SPI	1^#^	1^#^

*∗* indicates *P* < 0.05 versus Sham.

# indicates *P* < 0.05 versus IR.

## Abbreviations

IR: Ischemia reperfusion
MDA: Malondialdehyde
ROS: Reactive oxygen species
SD: Standard deviation
SPI: Spironolactone.
